# P-83. Corynebacterium Striatum, an emerging community and nosocomial pathogen

**DOI:** 10.1093/ofid/ofae631.290

**Published:** 2025-01-29

**Authors:** Dennys Jimenez, Jose Cadena-Zuluaga, Serene Hoskins, Kelly Reveles

**Affiliations:** DMG AZ, Phoenix, Arizona; University of Texas health and science center San Antonio, Audie L. Murphy VA Medical Center, San Antonio, Texas; UT Health Science Center San Antonio, San Antonio, Texas; The University of Texas at Austin, San Antonio, Texas

## Abstract

**Background:**

*Corynebacterium striatum* is part of the normal flora of the skin and nasal mucosa. Cases of *C. striatum* isolates are commonly neglected and assumed as contaminants, but increasing reports suggests it is the causative pathogen in many community and nosocomial infections. The objective of this study was to review and analyze *C. striatum* isolates from sterile sites over a 12-year period (2011 -2023)

Corynebacterium Striatum, an emerging community and nosocomial pathogen Table 1

**Figure:**
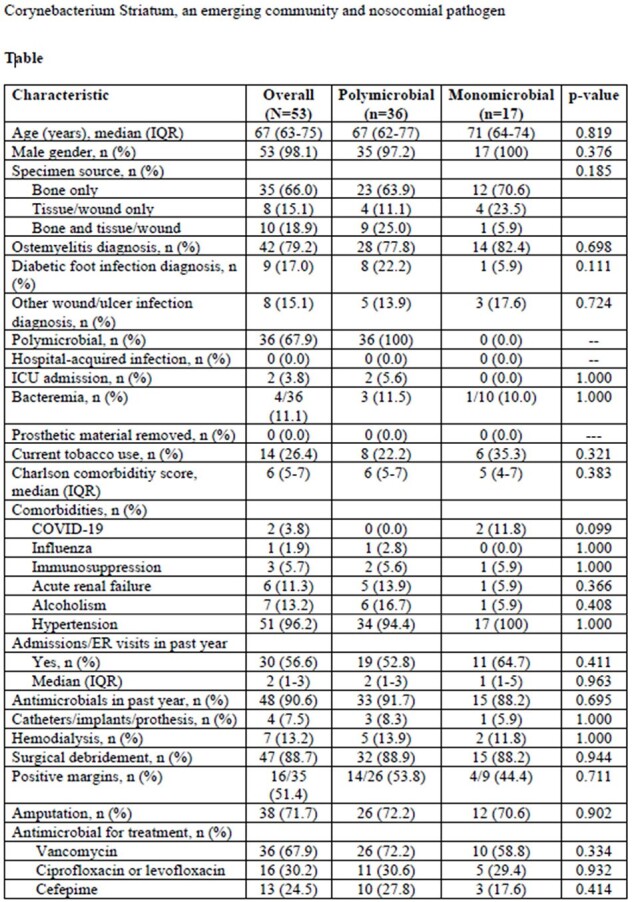

Patient's clinical characteristics divided by type of infection (Monomicrobial vs polymicrobial)

**Methods:**

This was a retrospective, observational study of patients with microbiologically confirmed *C. striatum* diabetic foot infection or osteomyelitis from July 1, 2011 to January 10, 2023 at the South Texas Veterans Health Care System. We screened all microbiology results using Theradoc (Premier Inc. Charlotte, NC) and reviewed the medical records of all patients 18 years or older that meet criteria. We collected epidemiological, radiological, laboratory and clinical data from each case. Additional variables included patient demographics, comorbidities (including Charlson comorbidity index), exposure to healthcare and antimicrobials within 12 months, presence of catheters or implants at the time of admission/infection, hemodialysis, treatment and outcomes. Variables were presented descriptively and compared between monomicrobial and polymicrobial infections using the chi-square or Wilcoxon rank sum test.

Corynebacterium Striatum, an emerging community and nosocomial pathogen Table 1 continuation

**Figure:**
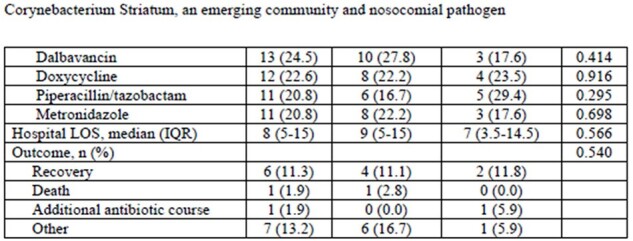

Patient's clinical characteristics divided by type of infection (Monomicrobial vs polymicrobial)

**Results:**

There were 185 potential candidates for inclusion, but 53 cases were included for analysis. Patient and infection characteristics are presented in the Table. Median age was 67 years and 98.1% were male. Most infections were osteomyelitis (79.2%) and polymicrobial (67.9%). Most patients had recent hospital admissions/ER visits (56.6%). and antimicrobial use (90.6%) within the last year. Polymicrobial and monomicrobial infections were similar. Antimicrobial susceptibility was available in 5 cases. All isolates were susceptible to vancomycin, daptomycin, and gentamycin. 4 were tested for linezolid and rifampin and they were all were susceptible. Amputation was frequently required in the management of infections (71.7% of cases).

**Conclusion:**

While relatively uncommon, *C. striatum* can lead to bone and wound infections that can result in poor patient health outcomes. Larger studies are needed to confirm the clinical relevance of this pathogen.

**Disclosures:**

**All Authors**: No reported disclosures

